# Strength and Bioelectrical Activity of the Pelvic Floor Muscles and Sexual Function in Women with and without Stress Urinary Incontinence: An Observational Cross-Sectional Study

**DOI:** 10.3390/healthcare11020181

**Published:** 2023-01-06

**Authors:** Clicia Raiane Galvão Ferreira, Wenderk Martins Soares, Caren Heloise da Costa Priante, Natália de Souza Duarte, Cleuma Oliveira Soares, Kayonne Campos Bittencourt, Giovana Salomão Melo, Erica Feio Carneiro Nunes, Fabiana de Campos Gomes, João Simão De Melo Neto, Cibele Nazaré Câmara Rodrigues

**Affiliations:** 1 Clinical and Experimental Research Unit of the Urogenital System (UPCEURG), Institute of Health Sciences, Federal University of Pará (UFPA), Belém 66075110, PA, Brazil; 2 CAFISIO Mulher, Belém 66075110, PA, Brazil; 3 State University of Pará (UEPA), Belém 66075110, PA, Brazil; 4 Ceres Faculty of Medicine (FACERES), São Paulo 15093-000, SP, Brazil

**Keywords:** pelvic floor, urinary incontinence, stress, sexual dysfunction, electromyography, quality of life

## Abstract

Stress urinary incontinence (SUI) results from an increase in intravesical pressure, which exceeds the pressure at which the urethra remains closed. Symptoms cause social and sexual intercourse discomfort directly or indirectly, which affect health-related quality of life and are associated with pelvic floor muscle (PFM) dysfunction. We aimed to verify the variation in strength and PFM bioelectrical activity and sexual function in women with SUI. Additionally, we analyzed the impact of this dysfunction on quality of life. This was an observational cross-sectional study. Women aged 25–55 years with frequent sexual intercourse were included. Women with SUI were included in a study group (G2, *n* = 17), and those without any type of incontinence were included in a control group (G1, *n* = 16). Primary outcomes were level of strength and PFM bioelectrical activity and sexual function as determinants of worse SUI in the control group. Secondary outcomes were associated between the primary outcomes and severity of urinary loss, impact on daily life, and quality of life in women with SUI. In the domains evaluated in the Female Sexual Function Index (FSFI), only sexual desire was lower in women with SUI (G2) than in the controls (*p* = 0.033). During analysis of G1 variables, a positive and moderate correlation was observed between power/myoeletric activation and maximum voluntary contraction (MVC) (*p* < 0.01), peak (*p* < 0.01), and mean amplitudes (*p* = 0.017). There was a high positive correlation between sexual arousal and other variables, including vaginal lubrication, sexual orgasm, and total FSFI value (*p* < 0.001 for all analyses). During evaluation of G2 variables, the MVC was positively correlated with the peak and mean amplitudes (*p* < 0.0001). Additionally, there was a high and positive correlation between the mean amplitudes (%MVC) and personal relationships (KHQ) (*p* = 0.001); the same was observed between the total (ICIQ) and activities of daily living (ICIQ) (*p* < 0.0001). Therefore, women with SUI presented with lower sexual desire and bioelectric activity but were not related to PFM strength. Additionally, the domains of sexual function and certain variables of quality of life are aggravated by SUI.

## 1. Introduction

Stress urinary incontinence (SUI) is defined as an involuntary and rapid loss of urine secondary to an increase in intra-abdominal pressure, which results in an increase in intravesical pressure that exceeds the maximum urethral closure pressure. Additionally, this loss is usually associated with vigorous physical activities, such as exercising, coughing, or sneezing, which cause discomfort or directly affect quality of life [1,2]. The etiology of SUI is multifactorial and is often associated with pelvic floor muscles (PFM) dysfunctions in the bladder and ligament structures, among others [1].

PFM consists of ligament components, fascias, and muscles that support the reproductive organs, bladder, and rectum. Thus, the proper functioning of these components enables opening and closing of structures, such as the vagina during childbirth, urethra during urination, and rectum during defecation. Myofascial disorders lead to PFM dysfunction, and as a consequence, muscle weakness and urinary incontinence [2]. In this sense, electromyography (EMG) is a supporting tool used to verify the bioelectrical activity of the PFM to contribute to the assessment of muscle function performance [3]. However, it is still not well established whether the subjective assessment of the level of strength performed manually by a professional is related to the bioelectrical activity of PFM.

Female sexual dysfunction is predominant in SUI, which is associated with urine loss, including penetration and reduction in sexual satisfaction, excitement, desire, and lubrication. Additionally, there is an increased risk of dyspareunia, vaginal dryness, and reduced libido [4]. This fact affects not only the sexual function of women with SUI but also their relationship with their partner, which may present a worse overall sexual experience, and consequently a decrease in quality of life [4,5]. Therefore, it is important to analyze the sexual function of women with SUI. However, there are few studies that have analyzed the relationship between bioelectric activity and sexual function in these women.

Additionally, questionnaires contribute to the diagnosis and severity of incontinence, its impact on sexual function, and general quality of life in women. Urinary incontinence and poor quality of life are two common associated conditions [1]. The main consequences of this association include social isolation, anxiety, depression, and reduced sexual activity. Although these questionnaires are frequently applied, this study has the potential for originality because of few studies that identify the assessment of these questionnaires against a technological resource with objective assessment.

Therefore, this study aimed to verify the variation in the level of strength and bioelectrical activity of the PFM and sexual function in women with SUI. Additionally, we analyzed the impact of this dysfunction on quality of life. In this context, the hypothesis formulated was that women with urinary incontinence will have lower muscle strength and PFM bioelectric activity, leading to impairments in sexual function and quality of life compared to women without SUI.

## 2. Materials and Methods

### 2.1. Study Design

This is an observational cross-sectional study with descriptive and inferential analysis.

### 2.2. Setting and Period of Study

This study was conducted using data from a private clinic in the area of women’s health in the city of Belém, PA, Brazil, in 2021.

### 2.3. Population

The study was conducted in women with or without SUI.

### 2.4. Eligibility Criteria

Women with SUI aged 25–55 years who had frequent sexual intercourse (defined in this study as ≥2 times a month) (2) were included. Those who presented with cognitive alterations, neurological injuries, pregnant and postpartum women, those who had undergone perineal reconstruction surgery, or who had another type of urinary incontinence were excluded.

### 2.5. Sampling

A non-probability type of convenience sampling was used to select the patients.

### 2.6. Sample

The sample considered a minimum of 22 participants based on the variable, “King’s Health Questionnaire (KHQ) score (part 2)”, determined randomly from the study by Chmielewska et al. (2019) [6], which offered an effect size of 1.30 with a *p* value of <0.05 for the two-tailed analysis. A minimum of 13 participants in group 1 (control) and 9 in group 2 (SUI) were considered based on the N2/N1 allocation ratio (0.72), with an α error of 0.05 and β of 0.2.

The initial sample comprised 34 women with or without SUI. One woman was excluded because she was aged >55 years. Therefore, 33 patients were selected based on the eligibility criteria. Women without SUI were considered controls (G1, *n* = 16), whereas a similar group of women with SUI was categorized as group 2 (G2, *n* = 17).

### 2.7. Data Collection and Variables

The patients were initially evaluated for incontinence by a physiotherapist. Later, another blinded professional (uni-blind) assessed the level of strength and bioelectrical activity of the PFM and sexual function. A form elaborated by the authors was used for data collection, including age, marital status, and whether women had sexual frequency greater than twice in the last month.

The International Consultation on Incontinence Questionnaire-Short Form (ICIQ-SF) and KHQ were used to assess urinary incontinence. Initially, oriented questions about the type of incontinence in the questionnaires were used as the inclusion criteria for SUI and exclusion criteria for the other types. These questions were not considered in the final score of these questionnaires. Women without incontinence were included in the control group.

### 2.8. Primary Outcomes

The primary outcome considered the level of strength and bioelectrical activity of the PFM and sexual function as determinants of worse SUI in the control group.

### 2.9. Secondary Outcomes

The secondary outcomes considered the association between the primary outcomes and severity of urinary loss, impact on daily life, and quality of life in women with SUI.

### 2.10. Level of Strength of the PFM

The muscle contraction capacity of the PFM was assessed using a perfect scheme [7], an evaluation method created to qualify the main components of pelvic muscle contractility. We used only the *p* component (power (pressure)), which refers to the measurement graduated with scores from 0 to 5 (0 = no contraction; 1 = flickerz; 2 = weak; 3 = moderate; 4 = good (with lift); and 5 = strong) of perineal muscle strength that was measured by digital palpation during a voluntary contraction and evaluated according to the modified Oxford grading system [8,9].

### 2.11. Bioelectrical Activity of the PFM

During evaluation of the bioelectric activity of the pelvic muscles, which was performed for electromyography (EMG), although it is a New Miotool EMG biofeedback of the Miotec (Miotec Equipamentos Biomédicos Ltd., Porto Alegre, RS, Brazil). This equipment is responsible for capturing muscular electrical activity in microvolts (µV), using an EMG sensor coupled to a vaginal probe and a reference electrode positioned on the patient’s lateral malleolus. Furthermore, the participants were instructed to remain supine [10]. However, adaptation to the gynecological position on the stretcher was performed. In sequence, the therapist’s verbal command was given to measure the maximum voluntary contraction (MVC) of the PFM, peak of contraction in a range of 0–10 s for raw and normalized data (%MCV), and the mean contraction amplitudes in a range of 0–10 s for raw and normalized data (%MCV).

### 2.12. Sexual Function

To assess sexual function, the Female Sexual Function Index (FSFI) was applied in the version translated, adapted, and validated for the Portuguese language [11]. This self-administered questionnaire contains 19 questions divided into the following domains: sexual desire, sexual excitement, vaginal lubrication, orgasm, sexual satisfaction, and pain. The rating lasted for four weeks. Each question ranges in score between 0 and 5, increasing in relation to the presence of the questioned function or in an inverted manner, as in the case of questions about pain.

### 2.13. Severity of Urinary Loss and Impact on Daily Life

To quantify urinary loss, the ICIQ-SF was used in the version translated and validated in Portuguese [12]. It has four questions to assess the frequency, severity, and impact of the severity of urinary loss. Sum of the first three questions defines the total score, and the cutoff point to be considered was eight points. Therefore, ≥8 points indicates that UI has a high impact, and <8 points represents a low impact. The impact on daily life was defined according to the score of question five: (0 points), none (1–3 points), mild (4–6 points), moderate (7–9 points), severe, and very severe (10 point) [13]. The questionnaire was applied to only G2.

### 2.14. Impact of SUI on Quality of Life

The KHQ was used in the version translated and validated in Portuguese Language [14]. The questionnaire was applied with the aim of evaluating the impact of SUI on quality of life. It comprises 30 questions that are distributed into nine domains: health perception, impact of incontinence, limitations of task performance, physical limitation, social limitation, personal relationship, emotions, sleep, disposition, and gravity. The quality of life score ranges between 0 and 100 with higher scores indicating worse quality of life. The questionnaire was applied to only G2.

### 2.15. Statistical Analysis

Descriptive statistical analysis was carried out using mean with standard deviation (parametric data) or medians with 95% confidence interval (CI) (non-parametric data) for each group. The normality of the data was analyzed using the Shapiro–Wilk test. For inferential analysis, the following tests were performed: non-paired t test and Mann–Whitney U test for intergroup comparisons analyzed with parametric and non-parametric distributions, respectively. Relationships between the variables were assessed using the Spearman’s rank (rs) (non-parametric data). Correlation was classified as null (<0.10), low (0.10–0.33), moderate (0.33–0.66), and high (>0.67).

## 3. Results

**Figure 1:**
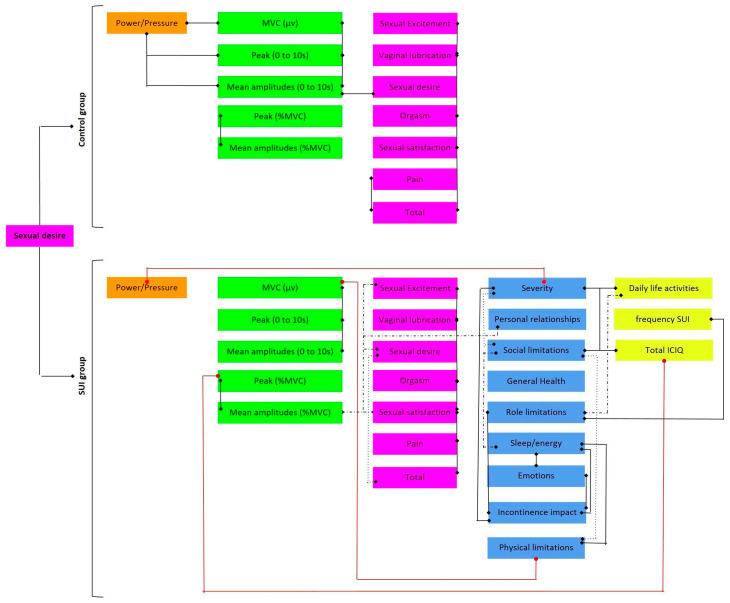
Schematic of results showing significant correlations in the control and SUI groups. Black lines represent positive correlations. Red lines represent negative correlations. Purple box: FSFI; Orange box: Power (pressure); Green box: sEMG; Blue box: KHQ; Yellow box: ICIQ.

This study analyzed women aged 25–55 years. The median age of the study population was 28 years (95% CI: 28–36 years). The study population was distributed according to marital status as single (G1:13; G2:10) or in a stable relationship (G1:3; G2:7) (Fisher’s exact test, *p* = 0.168). The results are shown in Figure 1.

### 3.1. Comparison among the Control and SUI groups

The level of strength and bioelectrical activity of the PFM and sexual function in women with SUI (G2) compared to the control (G1) are presented in Table 1. Sexual desire was lower in G2. No significant differences were found in the other variables.

### 3.2. Correlations among Variables in the Control Group

Table 2 shows the correlations between the variables in the control group. Thus, a moderate positive correlation was observed between the power (pressure) and MVC and peak and mean amplitudes. The MVC has a high positive correlation with the peak and mean amplitudes. The peak correlates highly and positively with the mean amplitudes (raw or normalized data). The mean amplitudes (% MVC) had a moderate positive correlation with the variable sexual desire (FSFI). There was a high and positive correlation between sexual excitement and the variables, including vaginal lubrication, sexual orgasm, and total FSFI value. Vaginal lubrification was high and positively correlated with orgasm variables and total FSFI score, but it had a moderate correlation with sexual satisfaction. Orgasm was shown to have a strong positive correlation with sexual satisfaction and total FSFI scores. Sexual satisfaction and pain showed strong and moderate positive correlations with the total FSFI score, respectively.

### 3.3. Correlations among Variables in the SUI Group

Table 3 shows the correlations between the variables in the SUI group. Power (pressure) correlated moderately and negatively with the KHQ severity measures.

Maximum voluntary contraction was high and positively correlated with the peak (0–10 s) and mean amplitudes (0–10 s), and it negatively correlated with the KHQ physical limitations. Furthermore, the peak (0–10 s) was strongly and positively correlated with the mean amplitudes (0–10 s). The peak (%MCV) correlated strongly and positively with the mean amplitudes (%MVC) and negatively and moderately with the total ICIQ total. The total ICIQ correlated positively with activities of daily living (ICIQ) (high correlation), social limitations (KHK), and severity measures (KHQ) (moderate correlation). A positive correlation was observed between the mean amplitudes (%MVC) and the following domains: sexual desire (FSFI), sexual excitement (FSFI), sexual satisfaction (FSFI) (moderate correlation), and personal relationships (KHQ) (high correlation).

The total (ICIQ) positively correlated with daily life activities (ICIQ) (high correlation), social limitations (KHK), and severity measures (KHQ) (moderate correlation). There was a moderate and positive correlation between the frequency (ICIQ) and role limitations (KHQ). Activities of daily living (ICIQ) showed moderate and positive correlations with the variable’s role limitations (KHQ), social limitations (KHQ), and severity measures (KHQ).

Sexual desire (FSFI) was moderately and positively correlated with sexual excitement (FSFI), sexual satisfaction (FSFI), and total (FSFI). However, its correlation with the general health perception (KHQ) was moderate and negative. It was observed that sexual excitement (FSFI) had a high and positive correlation with vaginal lubrication (FSFI), orgasm (FSFI), sexual satisfaction (FSFI), pain (FSFI), and total (FSFI). Vaginal lubrication (FSFI) with orgasm (FSFI), sexual satisfaction (FSFI), pain (FSFI), and total (FSFI) domains showed a high and positive correlation. Furthermore, orgasm (FSFI) is correlated with the domains of sexual satisfaction (FSFI), pain (FSFI), and total (FSFI). Furthermore, sexual satisfaction (FSFI) with pain (FSFI) and total (FSFI) variables were highly correlated.

The pain (FSFI) and total (FSFI) scores were strongly and positively correlated. Still, incontinence impact (KHQ) was strongly and positively correlated with emotions (KHQ) and sleep/energy (KHQ), while the domains of role limitations (KHQ) and severity measures (KHQ) have a moderate and positive correlation.

Physical limitations (KHQ) positively correlated with the social limitations (KHQ) (high correlation) and sleep/energy (KHQ) (moderate correlation). Furthermore, the correlation between social limitations (KHQ) was perceived as moderate and positive with sleep/energy (KHQ) and severity measures (KHQ). Finally, emotions (KHQ) were moderately and positively correlated with sleep/energy (KHQ).

## 4. Discussion

The present study evaluated the correlation between the level of contraction of the PFM, sexual function, and quality of life in women with and without urinary incontinence by effort.

A study by De Luccas et al. [15] on the functionality of PFM demonstrated that women with weak PFM strength were more likely to have urogynecological, coloproctological, and sexual dysfunctions. When evaluating the subjective strength of these muscles using the PERFECT scheme, both groups presented with grade three, suggesting moderate strength. However, there were no differences between the groups in the strength and bioelectrical activity of the PFM.

Electromyography was used to assess the bioelectrical activity and PFM contraction capacity. The study by Oleksy et al. [16] corroborates this study and complements that analyses with EMG have high reliability as an assessment tool and obtain important information about specific deficits of muscle contractions, such as decreased muscle strength, endurance, or coordination related to SUI.

Additionally, performing EMG using vaginal probes, as performed in this study, is in agreement with what Ribeiro et al. [17] demonstrated in their study, that as the PFM are deeper, the use of other electrodes, such as the surface, is not suitable because it is a record of activities in close muscles, such as the gluteus and obturator hip musculature.

The PFM must have a tension at rest (tone) and a muscle contraction and relaxation capacity (control), according to the variations of intra-abdominal and intravesical pressure (coordination) [18]. EMG measures the electrical potentials generated during muscle contraction and is an important test in various disorders of muscle tone [19,20]. In this study, we observed that the normalized peak of bioelectrical activity decreased with the severity of urinary loss. Therefore, these results show a reduction in these capacities in incontinent women.

Regarding the total FSFI score, there was no statistical difference between the groups, and both G1 and G2 had a score below the cut-off line, which was 26.55 according to the valuation established by the instrument, suggesting sexual dysfunction. However, we emphasize that women with incontinence had lower scores [21].

These findings are in agreement with that of other studies that have shown the prevalence of sexual dysfunction in women diagnosed with urinary incontinence [22]. Lower FSFI scores were related to the severity of incontinence, in addition to the symptoms affecting female sexuality, which may present as decreased libido, vaginal dryness, and dyspareunia, thereby generating psychological insecurity and affecting the quality of life of these women [23,24].

The findings of this study revealed that the domain of sexual desire was reduced in women with urinary incontinence (G2) compared to women without this clinical condition (G1). It is suggested that the presence of SUI was a factor that contributed to the decrease in sexual desire compared to those who do not have this clinical condition, which is in agreement with the study by Burzyński, 2021 [25], in which they found that women with SUI had lower scores in the domain valuation by the FSFI, corroborating the findings of this study.

Women with urinary incontinence also had lower values in several other domains of the FSFI, such as orgasm, vaginal lubrication, sexual satisfaction, and pain level. Previous studies have reported similar results. Renly et al. [3] showed that women with SUI had lower global sexual function, decreased sexual satisfaction, and a lower frequency of sexual intercourse with their partners, as urine loss may occur during sexual intercourse [26].

The quality of life of women with SUI is impaired by the difficulty in performing their daily activities, work relationships, and social relationships, such as going out with friends. Depending on the severity of urinary incontinence, such limitations experienced by these women become more evident [27]. Similar data were found in this research, which showed that the classification of the severity of urinary incontinence valuation by the ICIQ directly affects the daily life activities of these women.

## 5. Limitation of the Study

Regarding the limitations of this study, the low incidence of isolated SUI without other types of incontinence was a factor that restricted the selection of participants. In addition, the age range is large, and differences in women’s marital status can influence sexual function. In addition, there is a difference in the subjective assessment of strength with the oxford scale with that of electromyography, as it involves different muscles, since it is not possible to assess the same muscles in the two forms of evaluation in the study. Another limiting factor is the use of state-of-the-art technology, which involves high research costs. However, we performed sample calculations to ensure a minimum and sufficient sample size.

## 6. Conclusions

Women with SUI have lower sexual desire and bioelectrical activity with correlated parameters, but they are not related to strength, lower PFM strength impacting a worse severity of urinary loss, lowest normalized peak of bioelectric activity with worse severity of urinary loss and impact on daily life, and relationship between the domains of sexual function, except for sexual desire, which has specific characteristics and interferes with personal relationships. Additionally, other quality of life variables were simultaneously worsened by the presence of SUI, and such function can be influenced by social, emotional factors.

## Figures and Tables

**Table 1:** Level of strength and bioelectrical activity of the pelvic floor muscles and sexual function in women with stress urinary incontinence (G2) in relation to the control group (G1).

	G1 (*n* = 16)	G2 (*n* = 17)	t or U	* p *
Power (pressure)	3.00 (2.16; 3.46)	3.00 (2.59; 3.75)	114.00	0.414
sEMG				
MVC (µv)	34.69 (30.07; 47.97)	32.53 (24.80; 48.02)	117.00	0.494
Peak (0 to 10 s)	34.14 (29.99; 49.13)	26.86 (23.15; 45.98)	99.00	0.183
Peak (%MCV)	102.48 ± 12.90	96.36 ± 19.25	1.079	0.290
Mean amplitudes (0 to 10 s)	25.08 (20.74; 34.49)	18.44 (15.59; 31.03)	98.00	0.171
Mean amplitudes (%MVC)	71.98 ± 13.75	65.94 ± 13.58	1.269	0.214
FSFI				
Sexual desire	3.86 ± 0.87	3.13 ± 0.98	2.23	0.033
Sexual excitement	4.05 ± 1.46	3.32 ± 1.81	1.28	0.210
Vaginal lubrication	4.65 (3.35; 5.12)	3.90 (2.50; 4.95)	132.00	0.884
Orgasm	4.07 ± 1.61	3.38 ± 2.39	0.973	0.339
Sexual satisfaction	3.95 ± 1.63	4.05 ± 1.70	−0.167	0.868
Pain	4.20 (3.10; 4.73)	4.40 (2.29; 4.81)	133.00	0.914
Total	24.09 ± 6.71	21.17 ± 10.48	0.960	0.346

sEMG, surface electromyography; MCV, maximum voluntary contraction; FSFI, female sexual function index.

**Table 2:** Correlation among the control group variables (G1).

Variables		rs	*p*
Power (pressure)	MVC (µv)	0.652	0.006
	Peak (0 to 10 s)	0.653	0.006
	Mean amplitudes (0 to 10 s)	0.653	0.017
MVC (µv)	Peak (0 to 10 s)	0.985	<0.0001
	Mean amplitudes (0 to 10 s)	0.909	<0.0001
Peak (0 to 10 s)	Mean amplitudes (0 to 10 s)	0.909	<0.0001
Peak (%MCV)	Mean amplitudes (%MVC)	0.833	<0.0001
Mean amplitudes (%MVC)	Sexual desire (FSFI)	0.534	0.033
Sexual excitement (FSFI)	Vaginal lubrication (FSFI)	0.885	<0.0001
	Orgasm (FSFI)	0.766	0.001
	Sexual satisfaction (FSFI)	0.763	0.001
	Total (FSFI)	0.908	<0.0001
Vaginal lubrication (FSFI)	Orgasm (FSFI)	0.736	0.001
	Sexual satisfaction (FSFI)	0.517	0.040
	Total (FSFI)	0.859	<0.0001
Orgasm (FSFI)	Sexual satisfaction (FSFI)	0.668	0.005
	Total (FSFI)	0.850	<0.0001
Sexual satisfaction (FSFI)	Total (FSFI)	0.797	<0.0001
Pain (FSFI)	Total (FSFI)	0.644	0.007

rs, Spearman’s; MVC, maximum voluntary contraction; FSFI, female sexual function index.

**Table 3:** Correlation among variables in women with stress urinary incontinence (SIU) (G2).

Variables		rs	* p *
Power (pressure)	KHQ Severity measures	−0.491	0.045
MVC (µv)	Peak (0 to 10 s)	0.961	<0.0001
	Mean amplitudes (0 to 10 s)	0.954	<0.0001
	KHQ Physical limitations	−0.483	0.049
Peak (0 to 10 s)	Mean amplitudes (0 to 10 s)	0.978	<0.0001
Peak (%MCV)	Mean amplitudes (%MVC)	0.855	<0.0001
	ICIQ Total	−0.486	0.048
Mean amplitudes (%MVC)	Sexual desire (FSFI)	0.556	0.020
	Sexual excitement (FSFI)	0.500	0.041
	Sexual satisfaction (FSFI)	0.533	0.028
	Personal relationships (KHQ)	0.748	0.001
Total (ICIQ)	Daily life activities (ICIQ)	0.895	<0.0001
	Social limitations (KHQ)	0.482	0.050
	Severity measures (KHQ)	0.579	0.015
Frequency SUI (ICIQ)	Role limitations (KHQ)	0.578	0.015
Daily life activities (ICIQ)	Role limitations (KHQ)	0.578	0.015
	Social limitations (KHQ)	0.485	0.048
	Severity measures (KHQ)	0.515	0.035
Sexual desire (FSFI)	Sexual excitement (FSFI)	0.711	0.001
	Sexual satisfaction (FSFI)	0.625	0.007
	Total (FSFI)	0.628	0.007
	General health perception (KHQ)	−0.579	0.015
Sexual excitement (FSFI)	Vaginal lubrication (FSFI)	0.870	<0.0001
	Orgasm (FSFI)	0.803	<0.0001
	Sexual satisfaction (FSFI)	0.860	<0.0001
	Pain (FSFI)	0.782	<0.0001
	Total (FSFI)	0.943	<0.0001
Vaginal lubrication (FSFI)	Orgasm (FSFI)	0.781	<0.0001
	Sexual satisfaction (FSFI)	0.747	0.001
	Pain (FSFI)	0.919	<0.0001
	Total (FSFI)	0.936	<0.0001
Orgasm (FSFI)	Sexual satisfaction (FSFI)	0.794	<0.0001
	Pain (FSFI)	0.792	<0.0001
	Total (FSFI)	0.902	<0.0001
Sexual satisfaction (FSFI)	Pain	0.734	0.001
	Total (FSFI)	0.892	<0.0001
Pain (FSFI)	Total (FSFI)	0.917	<0.0001
Incontinence impact (KHQ)	Role limitations (KHQ)	0.535	0.027
	Emotions (KHQ)	0.756	<0.0001
	Sleep/energy (KHQ)	0.689	0.002
	Severity measures (KHQ)	0.413	0.100
Physical limitations (KHQ)	Social limitations (KHQ)	0.691	0.002
	Sleep/energy (KHQ)	0.603	0.010
Social limitations (KHQ)	Sleep/energy (KHQ)	0.615	0.009
	Severity measures (KHQ)	0.535	0.027
Emotions (KHQ)	Sleep/energy (KHQ)	0.487	0.047

rs, Spearman’s; MVC, maximum voluntary contraction; FSFI, female sexual function index.

## Data Availability

The data presented in this study are available at the request of the corresponding author. The data are not publicly available due to the privacy and ethics policy signed by the study participants.
